# Impact of prenatal, neonatal, and postnatal factors on epilepsy risk in children and adolescents: a systematic review and meta-analysis

**DOI:** 10.1186/s42494-023-00143-2

**Published:** 2024-01-02

**Authors:** Imen Ketata, Emna Ellouz, Rahil Mizouri

**Affiliations:** 1 Neurology Department, University Hospital of Gabes, Gabes, 6014 Tunisia; 2 Sfax University, Sfax, 3029 Tunisia; 3 Monastir University, Monastir, 5000 Tunisia

**Keywords:** Prenatal factors, Delivery factors, Postnatal factors, Epilepsy, Children, Adolescents

## Abstract

**Background:**

Epilepsy is a common, long-term neurological condition. Several previous case-control, cohort and cross-sectional studies have highlighted the role of prenatal, delivery and postnatal factors in the onset of epilepsy. In this systematic review, we evaluate the impact of these factors on the development of epilepsy in children and adolescents.

**Methods:**

We searched PubMed and Google Scholar for literature on the relationship between prenatal, delivery and postnatal factors and the occurrence of epilepsy. The research was performed according to the PRSIMA 2020 flowchart and checklist. Data were extracted and pooled according to the ReviewManager 5.3 software using a random-effects model. Sensitivity analysis and subgroup analysis were used to evaluate the source of heterogeneity.

**Results:**

We identified 25 reports, including 45,044 cases with confirmed epilepsy and 2,558,210 controls. Premature birth is significantly associated with the risk of epilepsy (pooled OR = 4.36 [95% CI: 1.26–15.09], *P* = 0.02). Smoking during pregnancy significantly increases this risk by 28% (pooled OR = 1.28 [95% CI:1.1–1.49], *P* = 0.002). Furthermore, maternal epilepsy confers a pooled OR of 2.06 [95% CI:1.26–3.36]. Eclampsia is linked to a 16.9-fold increased risk of epilepsy. In addition, both pregnancy metrorrhagia and maternal infection are significantly associated with the epilepsy risk (pooled OR = 2.24 [95% CI: 1.36–3.71] and 1.28 [95% CI: 1.17–1.41], respectively). For delivery conditions, cord prolapse (pooled OR = 2.58 [95% CI: 1.25–5.32]), prolonged labor (> 6 h) (OR = 6.74 [95% CI: 3.57–12.71]) and head trauma (pooled OR = 2.31 [95% CI: 1.54–3.48]) represent a meaningful risk of epilepsy occurrence. Moreover, birth complications (OR = 3.91 [95% CI: 2.43–6.29]), low birth weight (pooled OR = 1.83 [95% CI: 1.5–2.23]) and male birth (pooled OR = 1.18 [95% CI: 1.06–1.32]) are associated with an elevated risk of epilepsy in childhood and adolescence.

**Conclusions:**

Epilepsy in children and adolescents can be attributed to a multitude of intricate factors, notably those during pregnancy, delivery and the postnatal period. These findings highlight the crucial role of prenatal and postnatal care in reducing the impact of these factors on epilepsy occurrence.

**Supplementary Information:**

The online version contains supplementary material available at 10.1186/s42494-023-00143-2.

## Background

Epilepsy is a common chronic neurological disorder marked by a pathological tendency toward recurring and unprovoked seizures [1]. Epilepsy poses a burden for parents, children, and medical doctors. The annual incidence of epilepsy is 61.4 per 100,000 persons [2]. Notably, the incidence of epilepsy is highest in the first year of life, with a rate of ~ 150 cases/100,000 persons per year [3]. Additionally, the occurrence of repeated and unprovoked seizures in childhood reaches 0.8% by the age of 15 [3]. For those aged under 20, epilepsy can affect 1% of the population [4]. The etiology of epilepsy can be divided into three types: cryptogenic, symptomatic, and idiopathic [1]. Meanwhile, the underlying risk factors for epilepsy in childhood and adolescence vary from those associated with epilepsy in adults [1]. A risk factor for epilepsy is defined as a situation that increases the occurrence of epilepsy [5]. Although certain risk factors are well documented, such as infection in the central nervous system and metabolic disorders, others remain poorly understood, notably those associated with pregnancy characteristics [5]. In fact, 20% of epilepsy cases have no identifiable causes [6]. While extensive research has been conducted to understand the etiology and management of epilepsy, there is a growing interest in investigating the roles of prenatal factors, delivery conditions, and postnatal factors in the development and progression of epilepsy. Understanding the risk factors can help prevent epilepsy onset, decrease epilepsy prevalence in children and adolescents as well as its associated comorbidities, and aid healthcare professionals in identifying high-risk populations and making plausible preventive strategies. Hence, the impacts of prenatal factors, delivery condition and postnatal factors on epilepsy are still a subject of debate, with different studies yielding conflicting results. In this systematic review, we aim to establish the relationships of prenatal characteristics, newborn delivery situations and postnatal conditions with the risk of epilepsy.

## Methods

### Study design

This systematic review and meta-analysis was conducted following the guidelines of the Preferred Reporting Items for Systematic Reviews and Meta-Analyses (PRISMA) 2020 [7]. It has not been recorded or registered in any registry site. The checklist is provided as a supplementary material.

### Literature search

We searched for literatures on the relationship between perinatal/postnatal characteristics and the risk of epilepsy development in children and adolescents in PubMed and Google Scholar using the MeSH (Medical Subject Heading) terms with the assistance of the HeTOP site (https://www.hetop.eu/hetop/). The terms identified were combined by Boolean search operators, and we used the following phrases for the search: “Epilepsy” AND (“prenatal” OR “prenatal care” OR “prenatal injuries” OR “pregnancy” OR “postnatal” OR “postnatal care” OR “postpartum” OR “postpartum care” OR “postnatal injuries”) AND (“child” OR “children” OR “childhood” OR “adolescent” OR “infant” OR “adolescence”). We specifically looked for case-control, cohort and cross-sectional studies. The last search was made in September 2023. No language or date restrictions were set during the search. Only studies in humans and full-free papers were included. The title and abstract of the collected papers were reviewed by two investigators to assess whether the records covered risk factors for epilepsy, and then the full-texts were evaluated for eligibility. In the event of a disagreement, a third author would rejudge the article.

### Eligibility criteria

The inclusion criteria for literature are listed as below: (1) case-control, cohort or cross-sectional papers aiming to establish epilepsy risk factors in children or adolescents according to prenatal factors, newborn delivery conditions and postnatal characteristics; (2) free full-text; (3) reporting cases aged 0 to 20 years; (4) reporting epilepsy cases with normal birth without stroke, cerebral palsy, malformation or encephalopathy; and (5) reporting cases of confirmed epilepsy regardless of the seizure type (two or more unprovoked seizures or one seizure with abnormal electroencephalogram). Reports that included adult seizures or reported only one seizure with normal electroencephalogram or with hypoglycemia as a cause of neonatal seizure or febrile seizure were excluded.

We considered the following prenatal factors: maternal age, gestational age, maternal infection regardless of the type of infection, preeclampsia, gestational hypertension, gestational diabetes, eclampsia, smoking during pregnancy, maternal epilepsy, and pregnancy metrorrhagia regardless of the term. The factors that were considered to occur during newborn delivery conditions are as follows: cesarean section, forceps, breech presentation, cord prolapses, prolonged labor and meconium. Finally, the following factors were incorporated into the category of postnatal factors: birth complications (infection excluding nervous central system infection, respiratory distress, feeding or crying or breathing complications, Apgar < 6), male newborn (male gender), weight at birth under 2.5 kg and head trauma.

### Quality of studies

The validated Newcastle-Ottawa quality assessment scale (NOS) was applied to assess the quality of reports [2]. Two investigators independently screened the quality of articles based on three key aspects: (i) participant selection, (ii) comparability of groups, and (iii) determination of the exposure of interest for a case-control study and the outcome of interest for a cohort study.

### Data extraction

For each report, the following information was retrieved: first author, year of publication, country or region of the paper, study design, gender, number of cases, number of control groups, number of evaluated perinatal/postnatal factors in cases and controls and the odds ratio (OR) and 95% confidence interval (95% CI).

### Statistical analysis

The ReviewManager 5.3 software developed by the Cochrane Collaboration was used to analyze the data and pooled the odds ratio. For the pooled effect, statistical significance was set at *P* < 0.05. Meanwhile, when *P* = 0.05, we defined it as statistical significant when the 95% CI did not contain 0. OR was combined for dichotomous data and DerSimonian and Laird's general inverse variance technique [8] was used to estimate the between-study variance, in which each study's weight was inversely proportional to its variance. Given the possibility of high variability among studies due to differences in study origins and populations, we chose a random-effects model over a fixed-effects model. To analyze the heterogeneity across the different studies, the Cochran *Q* test (*P* < 0.1 was deemed significant) and *I*^*2*^ statistic were used. The heterogeneity was categorized as minimal (*I*^*2*^ value, 0–25%), low (25–50%), moderate (50–75%), and severe (> 75%). If considerable heterogeneity was found, a sensitivity analysis was performed by eliminating studies one by one to determine the likely causes of this heterogeneity. We ran a subgroup analysis if the sensitivity analysis failed to identify the source of heterogeneity. Because there were fewer than 10 combined studies in each risk factor analysis, it remained unnecessary to study the bias risk by funnel plot or Begg test, and Egger test [9, 10].

## Results

### Characteristics of the studies and evaluation of their quality

**Fig. 1:**
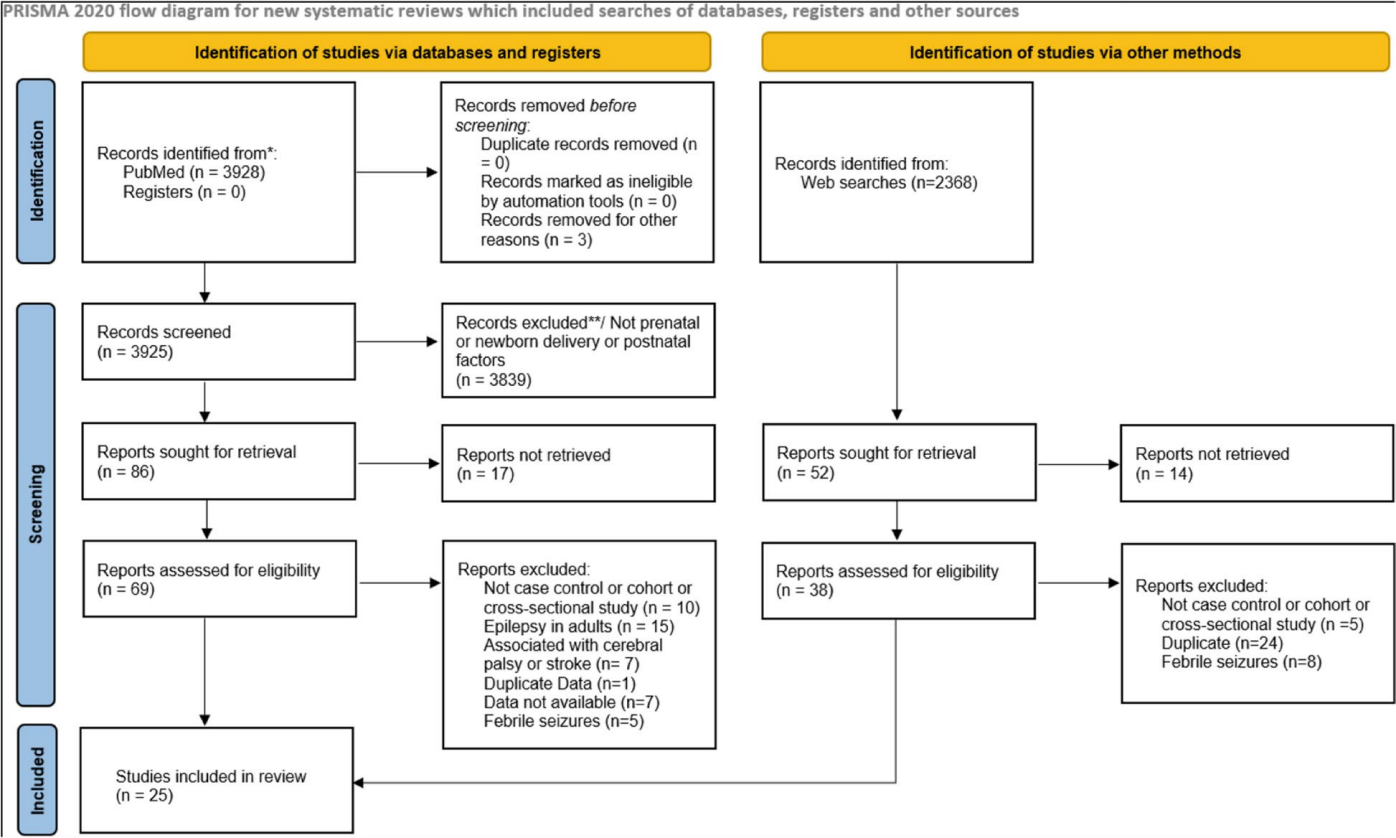
PRISMA flowchart showing the processes of literature search and screening for systematic review and meta-analysis

The initial search identified 6296 records from PubMed and Google Scholar. After screening the title, abstract, and full-text for eligibility, we identified 25 reports [5, 11–34]. Figure 1 illustrates the flowchart of the selection procedure. Overall, we collected 45,044 cases with confirmed epilepsy and 2,558,210 controls. Epilepsy was confirmed by clinical criteria (two or more unprovoked seizures) or one seizure coupled with an abnormal electroencephalogram.

Each of the case-control studies included was evaluated using the NOS. One study received a score of 4 and another had a score of 5. Seven reports had a score of 6, 11 had score of 7 and four had a score of 8. Only one study had a score of 9. Table 1 summarizes the features of the included studies.

**Table 1:** Characteristics of the studies included

Study	Country/Region	Study design	Epilepsy diagnosis	Patient with epilepsy (*n*)	Control without epilepsy (*n*)	NOS score
Henderson et al. (1964) [32]	United States	Case-control	Clinical^a^	283	283	6
Maheshwar et al. (1990) [31]	India	Cohort study	Clinical^a^	381	372	7
Kuenneth et al. (1996) [30]	United States	Case-control	Clinical^a^	78	408	7
Daoud et al. (2003) [5]	Jordan	Case-control	Clinical^a^	200	200	6
Asadi-Pooya et al. (2005) [14]	Iran	Case-control	Clinical^a^	142	138	6
Krebs et al. (2006) [17]	Denmark	Case-control	Unavailable	290	580	6
Mung’ala-Odera et al. (2008) [29]	United Kingdom	Case-control	Clinical^a^	110	816	8
Cansu et al. (2007) [23]	Turkey	Case-control	Clinical^a^	805	848	7
Masri et al. (2008) [28]	Jordan	Case-control	Seizure and EEG^b^	55	111	6
McDermott et al. (2010) [16]	United States	Case-control	ICD codes	2185	125,563	7
Attumalil et al. (2011) [25]	India	Case-control	Clinical^a^	82	160	4
Burton et al. (2012) [22]	Tanzania	Cross-sectional	Clinical^a^	112	113	8
Miller et al. (2012) [3]	Denmark	Cohort study	ICD codes	2848	444,781	7
Ngugi et al. (2013) [12]	Sub-Saharan Africa	Cross-sectional and case-control	Clinical^a^	825	1031	9
Sun et al. (2008) [13]	United States	Cohort study	Clinical^a^	664	89,973	8
Ae-Ngibise et al. (2015) [11]	Ghana	Cross-sectional and case-control	Clinical^a^	144	172	6
Cruz-Cruz (2017) [24]	Mexico	Case-control	Clinical^a^	118	118	6
Thygesen et al. (2018) [34]	Denmark	Cohort study	Clinical^a^	2326	92,700	7
Hirvonen et al. (2017) [18]	Finland	Cohort study	Seizure and EEG^b^	5611	1,027,738	7
Kakooza-Mwesige (2017) [27]	United Kingdom	Case-control	Clinical^a^	155	171	8
Odd et al. (2018) [19]	United Kingdom	Cohort study	ICD codes	4594	467,038	7
Chou et al. (2020) [21]	Taiwan region of China	Cohort study	ICD codes	21,474	94,720	7
Whitehead et al. (2006) [20]	Canada	Cohort study	Seizure and EEG^b^	648	124,207	7
Chowdhury et al. (2020) [26]	Bangladesh	Cross-sectional	Unavailable	55	55	5
Gumisiriza et el (2021) [33]	Belgium	Case-control	Clinical^a^	154	153	7
Total	-	-	-	45,044	2,558,210	-

^a^, Two unprovoked seizures; *NOS* Newcastle-Ottawa Scale, *ICD* International Classification of Diseases, *EEG* electroencephalogram

^b^, abnormal

### Meta-analysis

The number and the type of prenatal, newborn delivery and postnatal risk factors varied among studies. Some risk factors could not be pooled for analysis since their association with epilepsy was only reported in one study (alcohol consumption during pregnancy, vacuum extraction delivery, cephalic presentation and breastfeeding). Overall, we analyzed 20 factors (10 prenatal factors, 6 newborn delivery factors and 4 postnatal factors) for epilepsy occurrence in children or adolescents (age < 20).

### Prenatal factors

#### Gestational age (< 37 weeks or > 42 weeks)

**Fig. 2:**
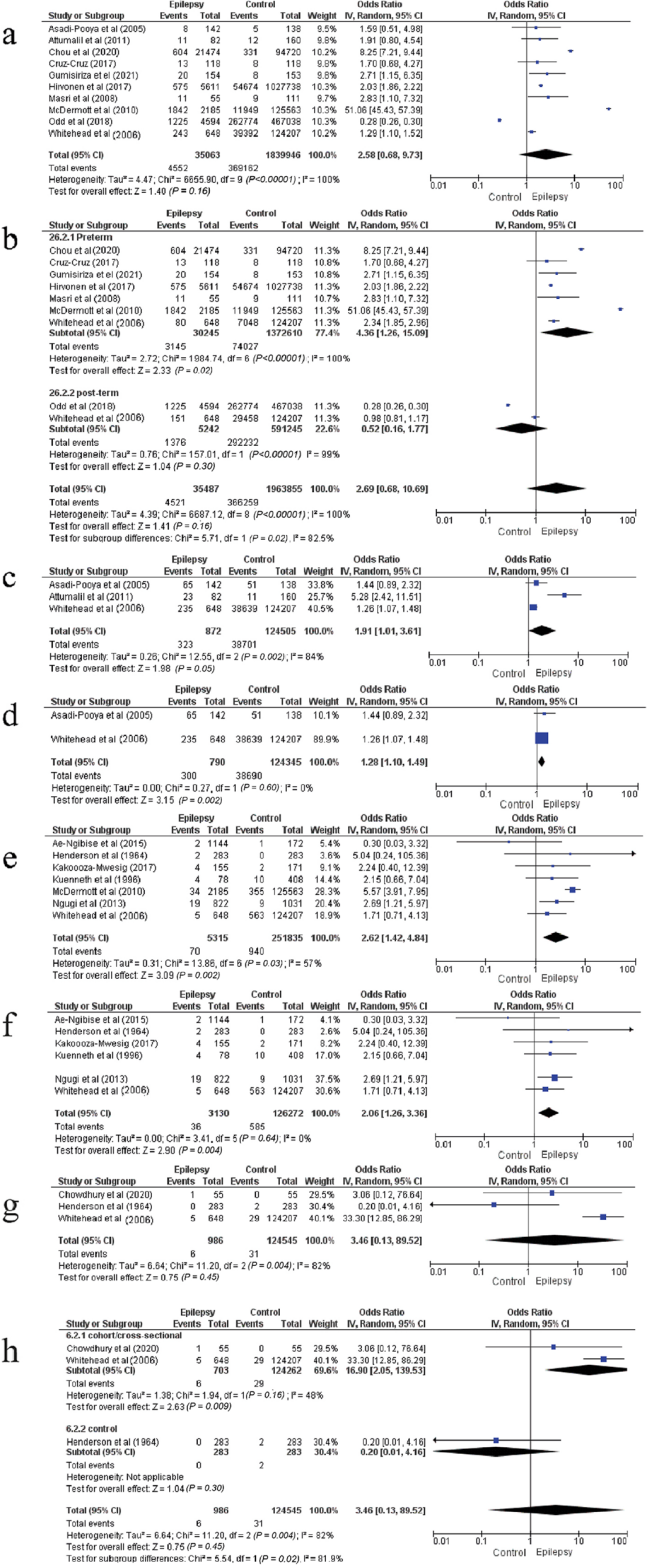
Forest plot of the associations of gestational age, smoking, maternal epilepsy and eclampsia with the epilepsy risk. **a** Forest plot of the association between gestational age and the epilepsy risk. **b** Forest plot of the association between gestational age and the epilepsy risk after subgroup analysis. **c** Forest plot of the association between smoking during pregnancy and the epilepsy risk. **d** Forest plot of the association between smoking during pregnancy and the epilepsy risk after sensitivity analysis. **e** Forest plot of association between maternal epilepsy and the epilepsy risk. **f** Forest plot of the association between maternal epilepsy and the epilepsy risk after sensitivity analysis. **g** Forest plot of the association between eclampsia and the epilepsy risk. **h** Forest plot of the association between eclampsia and the epilepsy risk after subgroup analysis

Gestational age < 37 weeks or > 42 weeks was not significantly associated with the risk of epilepsy in children or adolescents with significant heterogeneity (pooled OR = 2.58 [95% CI: 0.68–9.73], *P* = 0.16, *I*^*2*^ = 100%, Cochran's *Q* test < 0.00001) (Fig. 2a). Sensitivity analysis by excluding studies one-by-one showed no decrease in heterogeneity. Subgroup analysis revealed that premature birth was significantly associated with the risk of epilepsy (pooled OR = 4.36 [95% CI: 1.26–15.09], *P* = 0.02). Meanwhile, the heterogeneity was also significant (*I*^*2*^ = 100%, Cochran's *Q* test < 0.00001). Furthermore, postterm was not linked to the risk of epilepsy (OR = 0.52 [95% CI: 0.16–1.77], *P* = 0.3) (Fig. 2b). The study by Attumalil et al. was excluded because it combined preterm and postterm births into a single variable [25].

### Smoking

Smoking during pregnancy was associated with an increased prevalence of epilepsy in children or adolescents with significant heterogeneity (37% vs 31.1%, pooled OR = 1.91 [95% CI: 1.01–3.61], *P* = 0.05, *I*^*2*^ = 84%, Cochran's *Q* test = 0.002) (Fig. 2c). Meanwhile, after sensitivity analysis by excluding the study by Attumalil et al. [25], smoking during pregnancy was shown to significantly increase the risk of epilepsy in children or adolescents by 28%, with insignificant and negligible heterogeneity (38% vs 31.1%, pooled OR = 1.28 [95% CI:1.1–1.49], *P* = 0.002, *I*^*2*^ = 0%, Cochran's *Q* test = 0.6) (Fig. 2d).

### Maternal epilepsy

Maternal epilepsy significantly elevated the risk of epilepsy in children or adolescents, with significant heterogeneity (1.32% vs 0.4%, pooled OR = 2.62 [95% CI: 1.42–4.84], *P* = 0.002, *I*^*2*^ = 57%, Cochran's *Q* test = 0.03) (Fig. 2e). After sensitivity analysis by excluding the study by McDermott et al. [16], this risk factor was significantly associated (1.15% vs 0.5%) with the epilepsy occurrence (pooled OR = 2.06 [95% CI:1.26–3.36], *P* = 0.004, *I*^*2*^ = 0%, Cochran's *Q* test = 0.64) (Fig. 2f).

### Eclampsia

Eclampsia was not significantly associated with the risk of epilepsy in childhood or adolescence (0.6% vs 0.02%, pooled OR = 3.46 [95% CI: 0.13–89.52], *P* = 0.45, *I*^*2*^ = 82%, Cochran's *Q* test = 0.004) (Fig. 2g). Sensitivity analysis by excluding studies one-by-one did not show a decrease in heterogeneity. Subgroup analysis revealed that study design (cohort study/cross-sectional study or case‒control study) represented the source of heterogeneity (*I*^*2*^ = 81.9%, Cochran's *Q* test = 0.02). Additionally, subgroup analysis showed that eclampsia was associated with a 16.9- fold increase of the risk of epilepsy in children/adolescents with insignificant heterogeneity in cohort/cross-sectional studies (prevalence of epilepsy, 0.85% vs 0.02%, pooled OR = 16.9 [95% CI: 2.05–139.53], *P* = 0.009, *I*^*2*^ = 48%, Cochran's *Q* test = 0.16) (Fig. 2h).

### Pregnancy metrorrhagia

**Fig. 3:**
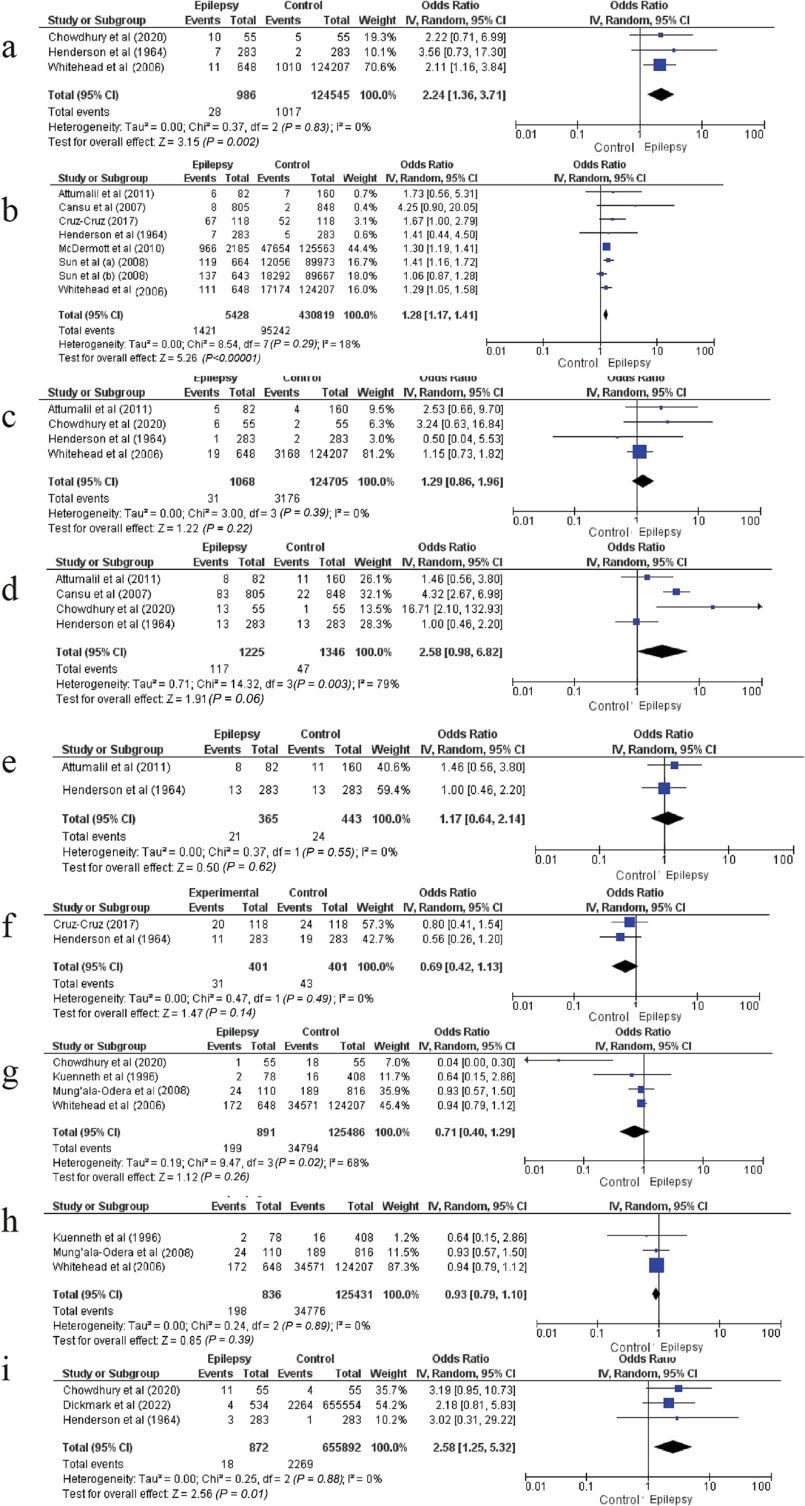
Forest plot of the associations of pregnancy metrorrhagia, infection, preeclampsia, gestational diabetes and hypertension, maternal age, and cord prolapse with the epilepsy risk. **a** Forest plot of the association between pregnancy metrorrhagia and the risk of epilepsy. **b** Forest plot of the relationship between maternal infection and the risk of epilepsy in children or adolescents. **c** Forest plot of association between gestational diabetes and the epilepsy risk. **d** Forest plot of the association between gestational hypertension and the epilepsy risk. **e** Forest plot of the association between gestational hypertension and the epilepsy risk after sensitivity analysis. **f** Forest plot of the association between preeclampsia and the epilepsy risk. **g** Forest plot of the association between maternal age and the epilepsy risk. **h** Forest plot of the association between maternal age and the epilepsy risk after sensitivity analysis. **i** Forest plot of the association between cord prolapse and the epilepsy risk

Pregnancy metrorrhagia, regardless of the term, was significantly associated with the risk of epilepsy occurrence in children or adolescents (prevalence of epilepsy, 2.8% vs 0.8%, pooled OR = 2.24 [95% CI: 1.36–3.71], *P* = 0.002, *I*^*2*^ = 0%, Cochran's *Q* test = 0.83). The heterogeneity was low and insignificant (Fig. 3a).

### Maternal infection

Maternal infection was shown to raise the risk of developing epilepsy by 28% with negligible and insignificant heterogeneity (26.2% vs 22.1%, pooled OR = 1.28 [95% CI: 1.17–1.41], *P* < 0.001, *I*^*2*^ = 18%, Cochran's *Q* test = 0.29) (Fig. 3b).

### Other factors

Gestational diabetes (Fig. 3 c), hypertension (Fig. 3d,e), preeclampsia (Fig. 3f), and maternal age (Fig. 3g,h) were not related to an increased risk of epilepsy in childhood or adolescence.

### Newborn delivery factors

#### Cord prolapse

Children/adolescents with epilepsy had a significantly higher prevalence of cord prolapse than the control groups (2.1% vs 0.3%) (pooled OR = 2.58 [95% CI: 1.25–5.32], *P* = 0.01, *I*^*2*^ = 0%, Cochran's *Q* test = 0.88) (Fig. 3i).

### Prolonged labor > 6 h

**Fig. 4:**
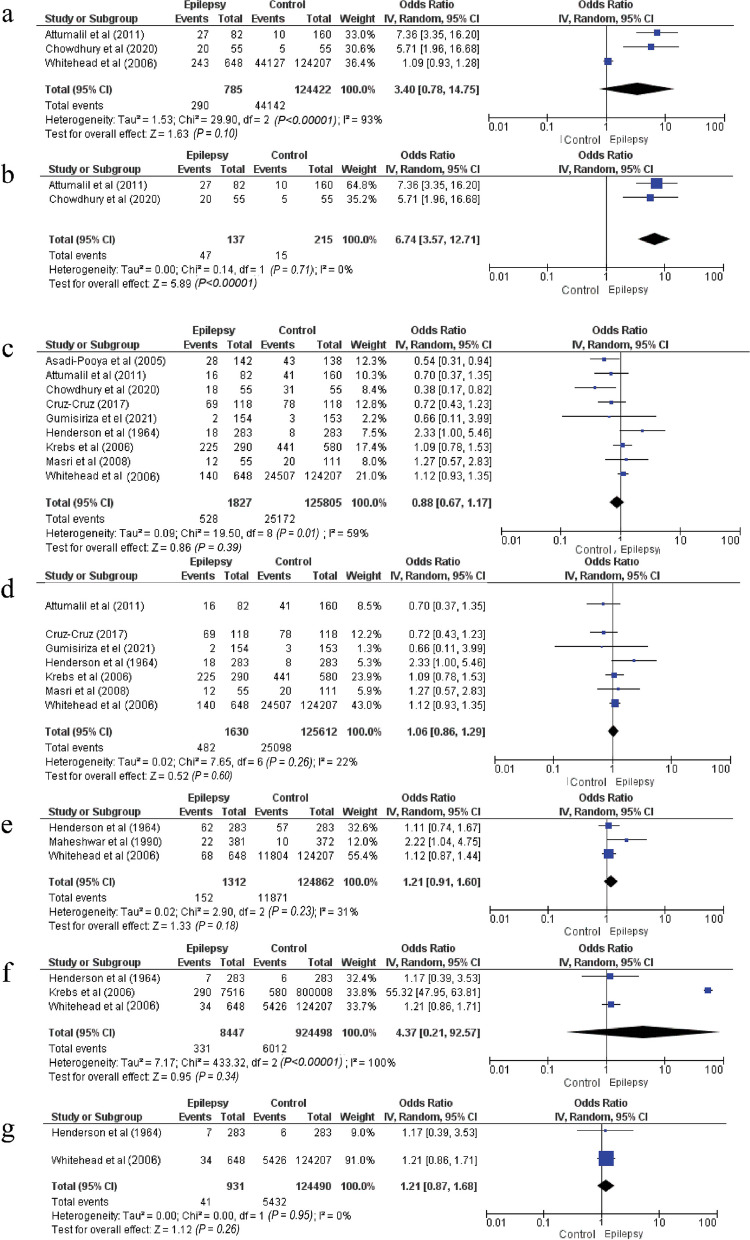
Forest plot of the associations of prolonged labor, cesarean section, forceps use, and breech presentation with the risk of epilepsy. **a** Forest plot of the association between prolonged labor and the epilepsy risk. **b** Forest plot of the association between prolonged labor and the epilepsy risk after sensitivity analysis. **c** Forest plot of the association between cesarean section and the epilepsy risk. **d** Forest plot of the association between cesarean section and the epilepsy risk after sensitivity analysis. **e** Forest plot of the association between forceps use and the epilepsy risk. **f** Forest plot of the association between breech presentation and the epilepsy risk. **g** Forest plot of the association between breech presentation and the epilepsy risk after sensitivity analysis

Prolonged labor > 6 h was not significantly connected to the risk of epilepsy in childhood or adolescence with significant heterogeneity (37% vs 35.5%, pooled OR = 3.4 [95% CI: 0.78–14.75], *P* = 0.1, *I*^*2*^ = 93%, Cochran's *Q* test < 0.00001) (Fig. 4a). Sensitivity analysis by eliminating the study of Whitehead et al. [20] revealed a significant decrease in heterogeneity (*I*^*2*^ = 0%, Cochran's *Q* test = 0.71). Prolonged labor (> 6 h) was associated with a 6.74-fold increase of epilepsy risk (34.3% vs 7% pooled OR = 6.74 [95% CI: 3.57–12.71], *P* < 0.001, *I*^*2*^ = 0%, Cochran's *Q* test = 0.71) (Fig. 4b).

### Other factors

**Fig. 5:**
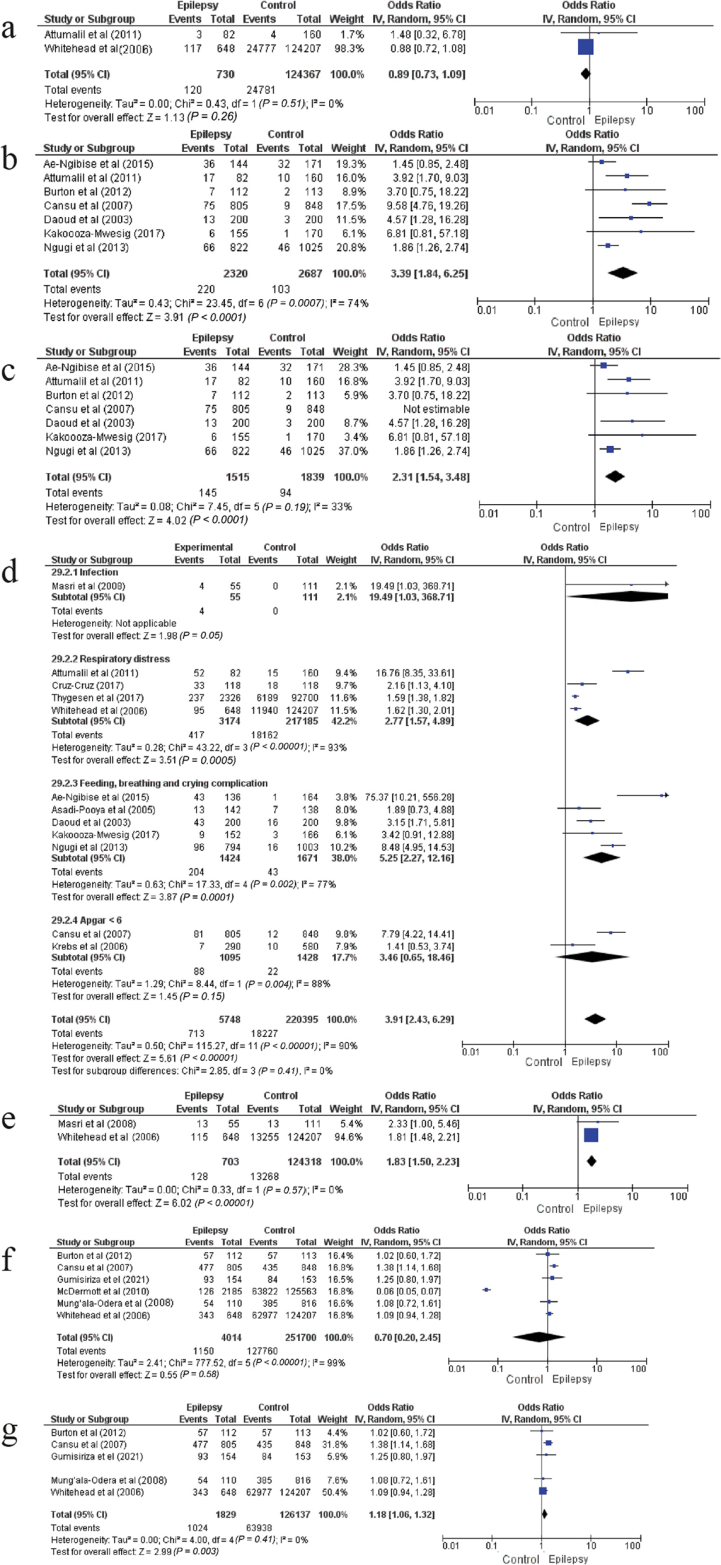
Forest plots of the associations of meconium, head trauma, birth complications, low birth weight and male gender with the epilepsy risk. **a** Forest plot of the association between meconium and the epilepsy risk. **b** Forest plot of the association between head trauma and the epilepsy risk. **c** Forest plot of the association between head trauma and the epilepsy risk after sensitivity analysis. **d** Forest plot of the association between birth complications and the epilepsy risk after subgroup analysis. **e** Forest plot of the association between low birth weight and the epilepsy risk. **f** Forest plot of the association between epilepsy risk and male newborns **g** Forest plot of the association between male gender and the epilepsy risk after sensitivity analysis

Cesarean section (Fig. 4c, d), forceps (Fig. 4e), breech presentation (Fig. 4f, g) and meconium (Fig. 5a) were not associated with the risk of epilepsy in children or adolescents.

### Postnatal factors

#### Head trauma

Head trauma was correlated with an elevated likelihood of epilepsy in children or adolescents (9.5% vs 3.8%, pooled OR = 3.39 [1.84–6.25], *P* < 0.001, *I*^*2*^ = 74%, Cochran's *Q* test = 0.0007) (Fig. 5b). The sensitivity analysis showed that after excluding the study by Cansu et al. [23], there remained a significant association between head trauma and epilepsy during childhood or adolescence, but with insignificant heterogeneity (9.5% vs 5.1%, pooled OR = 2.31 [95% CI: 1.54–3.48], *P* < 0.001, *I*^*2*^ = 33%, Cochran's *Q* test = 0.19) (Fig. 5c).

### Birth complications

Birth complications including feeding complications, crying, respiratory complications, infection (excluding central nervous system infection) and Apgar < 6, were significantly associated with a higher risk of epilepsy (12.4% vs 8.3%, pooled OR = 3.91 [95% CI: 2.43–6.29], *P* < 0.001). Meanwhile, the heterogeneity was high and significant (*I*^*2*^ = 90%, Cochran's *Q* test < 0.00001). Sensitivity analysis did not show any decrease in heterogeneity. Subgroup analysis showed that the complication type (infection, respiratory distress) was not the source of heterogeneity across studies (*I*^*2*^ = 0%, Cochran's *Q* test = 0.41). Figure 5d shows that the most significant risk factor was infection (OR = 19.49 [95% CI: 1.03–368.71], *P* = 0.05]).

### Low birth weight (< 2.5 kg)

Low birth weight significantly increased the risk of epilepsy in childhood or adolescence by 83% (18.2% vs 10.7%, pooled OR = 1.83 [95% CI: 1.5–2.23], *I*^*2*^ = 0%, Cochran's *Q* test = 0.57) (Fig. 5e).

### Gender

The male gender was not associated with the risk of epilepsy with high heterogeneity (28.6% vs 50.7%, pooled OR = 0.7 [95% CI: 0.2–2.45], *P* = 0.58, *I*^*2*^ = 99%, Cochran's *Q* test < 0.00001) (Fig. 5f). After sensitivity analysis by excluding the study by McDermott et al. [16], this risk factor was significant with negligible and insignificant heterogeneity (56% vs 50.7%, pooled OR = 1.18 [95% CI: 1.06–1.32], *P* = 0.003, *I*^*2*^ = 0%, Cochran's *Q* test = 0.41) (Fig. 5g).

Table 2 provides a concise overview of the variables associated with epilepsy occurrence in childhood and adolescence, categorized by prenatal, delivery, and postnatal periods.

**Table 2:** Summary of related and non-related variables for epilepsy onset in childhood and adolescence

Prenatal conditions			
Variables	Related	Non-related	Pooled OR [95% CI]
Preterm birth	×		4.36 [95% CI: 1.26–15.09]
Post-term birth		×	0.52 [95% CI: 0.16–1.77]
Smoking during pregnancy	×		1.28 [95% CI:1.1–1.49],
Maternal epilepsy	×		2.06 [95% CI:1.26–3.36]
Eclampsia	×		16.9 [95% CI: 2.05–139.53]
Pregnancy metrorrhagia	×		2.24 [95% CI: 1.36–3.71]
Maternal infection	×		1.28 [95% CI: 1.17–1.41]
Gestational diabetes		×	1.29 [95% CI: 0.86–1.96]
Hypertension		×	1.17 [95% CI: 0.64–2.14]
Preeclampsia		×	0.69 [95% CI: 0.42–1.13]
Maternal age		×	0.93 [95% CI: 0.79–1.10]
Newborn delivery conditions			
Variables	Related	Non-related	Pooled OR [95% CI]
Cord prolapses	×		2.58 [95% CI: 1.25–5.32]
Prolonged labor > 6 h	×		6.74 [95% CI: 3.57–12.71]
Cesarean section		×	1.06 [95% CI: 0.86–1.29]
Forceps		×	1.06 [95% CI: 0.86–1.29]
Breech presentation		×	1.21 [95% CI: 0.87–1.68]
Meconium		×	0.89 [95% CI: 0.73–1.09]
Postnatal factors			
Variables	Related	Non-related	Pooled OR [95% CI]
Head trauma	×		2.31 [95% CI: 1.54–3.48]
Birth complications	×		3.91 [95% CI: 2.43–6.29]
Low birth weight (< 2.5 kg)	×		1.83 [95% CI: 1.5–2.23]
Gender	×		1.18 [95% CI: 1.06–1.32]

*OR* odds ratio, *CI* confidence interval

## Discussion

The present systematic review and meta-analysis is the first to evaluate factors associated with epilepsy occurrence in childhood or adolescence. Meta-analysis based on the 25 studies revealed that the prenatal risk factors for epilepsy included preterm birth (< 37 weeks), smoking during pregnancy, maternal epilepsy, eclampsia, pregnancy metrorrhagia regardless of the term, and maternal infection regardless of the term. Newborn delivery factors that increase the risk of epilepsy were cord prolapse, head trauma and prolonged labor > 6 h. Regarding postnatal factors, the risk of epilepsy was significantly elevated with birth complications, low-weight birth (< 2.5 kg), and male gender.

Prenatal factors were more commonly studied in various papers [16, 18–21, 28, 33]. Of the studies addressing the relationship between preterm/postterm birth and the risk of epilepsy, we identified six case-control studies [14, 16, 24, 25, 28, 33] and four cohort studies [19–21, 33]. It is worth noting that these studies had comparable weights in terms of their impact on the overall results of the meta-analysis. Subgroup analysis and sensitivity analysis indicated that preterm birth doubled the risk of epilepsy in children or adolescents. The study conducted by Li et al. was in line with our findings, in which the risk was 2.16 times higher in preterm groups [35]. However, post-term birth was not associated with the risk of epilepsy. Several studies have indicated the association between preterm birth and epilepsy onset at a younger age is mediated by white matter gliosis and hypoxic-ischemic brain injury [13, 36]. However, in our study, by excluding cerebral palsy and stroke, we emphasize alternative hypotheses. In fact, hippocampal sclerosis, impaired development of brain structure and a higher risk of infection in preterm groups have been reported to contribute to this association [13]. Given the limited data on the risk of epilepsy occurrence in children and adolescents with preterm, full-term, or post-term births, we were unable to compare the risk of epilepsy between those with preterm and full-term births, and between those with postterm and full-term births.

Our results showed that smoking during pregnancy significantly increased epilepsy occurrence by 28%. Smoking during pregnancy has been identified as the first environmental risk factor for epilepsy worldwide. Furthermore, it doubles the risk of seizures in children [37]. Among the few studies exploring the association of smoking at pregnancy with epilepsy onset at an early age, smoking has been found to induce placental inflammation, placenta damage, decreased blood flow in the placenta, remodeling of the uterine vasculature, low birth weight and fetal growth restriction [38–40], which may underlie the increased risk of epilepsy [38–40]. Additionally, tobacco contains various chemicals with proconvulsant effects such as ammonia, hexane, toluene and arsenic; however, it remains unknown whether these chemicals can cross the placental barrier to induce epileptic seizures in children [40]. Furthermore, some studies have reported brain structural changes associated with maternal smoking, such as cortical thinning in the lateral and perisylvian occipital cortices and a significantly smaller frontal lobe [40, 41]. Moreover, maternal smoking affects the expression of many genes that are related with epilepsy [42]. Further research is required to elucidate the pathophysiology of smoking during pregnancy and the risk of epilepsy in children or adolescents.

In our study, children/adolescents born to epileptic mothers were 2.06 times more likely to have epilepsy. Out of the 6 studies that examined the association of maternal epilepsy with epilepsy onset in children or adolescents, two were cross-sectional and case-control studies [11, 12], three were case-control studies [27, 30, 32] and one was a cohort study [20]. Notably, the two studies that exerted the most significant influence on the overall results were those by Ngugi et al. (2013) and Whitehead et al. (37.5% and 30.6% respectively) [12, 20]. This finding was similar to those reported in various other studies [12, 16, 43]. In fact, maternal epilepsy was associated with an ~ 45% increased risk of epilepsy in the offspring [43]. Additionally, antiepileptic drug use during pregnancy is not an explanation for epilepsy occurrence in children/adolescents [44]. Another hypothesis that can explain this relationship is the genetic origin of epilepsy. In fact, epilepsy can be inherited from the mothers [43]. Additional studies are needed to fully establish the mechanism behind the association between maternal epilepsy and the risk of epilepsy in children and adolescents.

While preeclampsia was not identified as a risk factor for epilepsy, eclampsia was strongly associated with this risk and conferred an OR of 16.9. The variation in study design has been identified as the origin of heterogeneity in this analysis. Specifically, we integrated two cohort studies [19, 20] along with one case-control study [32]. The research conducted by Whitehead et al. carried the greatest significance in the analysis, accounting for 40.1% of the total weight [20]. Eclampsia is a serious complication that can lead to epilepsy in children/ adolescents through various pathways. In fact, mothers can experience hypoxia, which can affect the normal development of the fetal brain and increase the risk of epilepsy. Rocca et al. showed that eclampsia increases the risk of generalized tonic-clonic seizures, partial seizures and absence seizures by 2 folds; however, this relationship is not significant [45]. This association may be explained by placental dysfunction, biological changes during eclampsia, premature birth and low-weight birth. Meanwhile, the exact mechanism remains unknown. As preeclampsia precedes eclampsia, prompt diagnosis and treatment of preeclampsia are needed to prevent eclampsia onset and its associated complications.

In our meta-analysis, we found that maternal infection, regardless of the type or term, is the leading cause of epilepsy in children and adolescents. Sun et al. showed that prenatal exposure to maternal cystitis, pyelonephritis, persistent diarrhea, coughing, and vaginal yeast infections is linked to an elevated risk of juvenile epilepsy [13]. Additionally, Casetta et al. found that maternal illness, notably upper respiratory infections, is linked to an elevated incidence of cryptogenic and idiopathic partial epilepsy [46]. While the pathophysiological mechanisms remain unclear, a plausible explanation is that the immune response and cytokines might potentially induce placental abnormalities and fetal brain damage. This finding highlights the importance of antenatal care to prevent infection and other pregnancy-related issues. Other factors related to epilepsy occurrence are prolonged labor and cord prolapse. These results could also be explained by the increased risk of infection. Cord prolapses can also lead to oxygen deprivation inducing brain injury [47]. Glass et al. showed a positive association between cord prolapses and seizure occurrence in children with an OR of 6.9 [95% CI: 5.9–8.1] [47].

The occurrence of epilepsy following head trauma has been extensively studied in both children/adolescents and adults [45, 48]. We found that children/adolescents with a history of head trauma had a 2.31-fold increased risk of epilepsy. This finding aligns with the results of previous studies. In fact, this association can be linked to neuroinflammation, glial scars and brain injuries induced by head trauma.

After sensitivity analysis by excluding the study of McDermott et al. [16], the risk of epilepsy occurrence was shown to be higher in male newborns (56% vs 50.7%, pooled OR = 1.18 [95% CI: 1.06–1.32]). We excluded this particular study because it was the primary source of variations of results. Our findings are consistent with those of other studies [49, 50]. Two potential explanations for our findings are as follows: sex differences in cerebral connectivity and in astrocyte structure [51–53]. The male brain typically has a larger amygdala and thalamus, while the female brain features a larger hippocampus, caudate nuclei, regional gray matter, and cortices [54]. Studies have shown that men exhibit stronger right-side connectivity in the amygdala, while women display more prominent left-side connections [54]. These sex-related distinctions in brain development, influenced by steroid hormones, impact the susceptibility to seizures [54]. Additionally, astrocyte structural variations may contribute to the sex difference in epilepsy, as cultured astrocytes and microglia from male and female rats display distinct functional responses and inflammatory marker expression [52, 53]. Further research is needed to uncover the structural and neuroendocrine factors contributing to the sex differences in epilepsy.

In our study, birth complications including feeding complications, crying, respiratory complications, infection (excluding central nervous system infection) and Apgar < 6, are significantly associated with a higher risk of epilepsy occurrence (pooled OR = 3.91 [95% CI: 2.43–6.29]). This discovery aligns with previous research. Indeed, the presence of respiratory complications and an Apgar score below 6 increase the likelihood of neurodevelopmental issues and the risk of epilepsy development [55]. Hypoxia can result in energy depletion, oxidative stress, and inflammation, ultimately causing cellular death, which can contribute to the development of cerebral palsy and epileptic lesions [56]. Additionally, Frederik et al. discovered that the likelihood of an epilepsy diagnosis is elevated not only after central nervous system infections but also after a wide variety of peripheral infections [57]. Some infections can enhance the likelihood of experiencing seizures, particularly in individuals who already have a pre-existing susceptibility to epilepsy [58]. Infections, particularly those linked to inflammation, have the potential to alter the immune responses in the brain and disrupt the equilibrium of neurotransmitters [59], which may potentially elevate the likelihood of epilepsy development [58]. Conversely, adequate nutrition is essential for healthy brain development. Insufficient intake of vital nutrients can have adverse effects on brain growth and development, increasing the vulnerability to various neurological disorders, including epilepsy [59]. Moreover, difficulties with feeding can give rise to metabolic imbalances, such as hypoglycemia or disruptions in electrolytes. These metabolic irregularities can impact brain function and potentially provoke seizures [59].

We assume that multiple factors during the prenatal, delivery and postnatal periods interact synergistically and dynamically, elevating the risk of epilepsy in children or adolescents. For instance, eclampsia can contribute to premature birth and low-weight birth, which, in turn, can lead to frequent newborn infections and complications. Additionally, maternal infection can be linked to cord prolapse and prolonged labor. A better understanding of these factors is critical for advancing effective preventive and treatment measures to reduce the likelihood of epilepsy.

While our study is novel and represents the first meta-analysis on prenatal, delivery and postnatal factors, it has some limitations. First, our research included 25 studies with various study designs. Second, some data were unavailable, such as the exact term and the quantity or severity of pregnancy metrorrhagia, which prevented us from establishing a stronger relationship between these factors and epilepsy onset. Furthermore, the inclusion and exclusion criteria varied across the studies. The age of children/adolescents included in different studies ranged from 0 to 20 years, potentially introducing bias. Third, our study only examined children and adolescents, excluding adults. It is essential to acknowledge that the factors we examined may have implications for epilepsy in adulthood [60, 61].

## Conclusions

Epilepsy onset in children or adolescents is related to multiple and complex factors, according to the pregnancy and postnatal characteristics. Among these factors, eclampsia is the strongest prenatal risk factor, prolonged labor is the strongest delivery factor and child infection is the most influential postnatal factor. These findings call for improved awareness about these factors. Further studies are required to understand the physiological mechanisms underlying each of these factors.

## Supplementary Information

**Additional file 1.**

## Data Availability

Data are available from the corresponding author upon reasonable request.

## Abbreviations

NOS: Newcastle-Ottawa quality assessment scale
OR: Odds ratio
PRISMA: Preferred Reporting Items for Systematic Reviews and Meta-Analyses

## References

[1] Fisher RS, Acevedo C, Arzimanoglou A, Bogacz A, Cross JH, Elger CE, et al. ILAE official report: a practical clinical definition of epilepsy. Epilepsia. 2014;55:475–82. 24730690 10.1111/epi.12550

[2] Beghi E. The epidemiology of epilepsy. Neuroepidemiology. 2020;54:185–91. 31852003 10.1159/000503831

[3] Miller N, Ehrenstein V, Nielsen RB, Bakketeig LS, Sørensen HT. Maternal use of antibiotics, hospitalisation for infection during pregnancy, and risk of childhood epilepsy: a population-based cohort study. PLoS ONE. 2012;7:e30850. 22295115 10.1371/journal.pone.0030850 PMC3266299

[4] Christensen J, Vestergaard M, Pedersen MG, Pedersen CB, Olsen J, Sidenius P. Incidence and prevalence of epilepsy in Denmark. Epilepsy Res. 2007;76:60–5. 17686613 10.1016/j.eplepsyres.2007.06.012

[5] Daoud AS, Batieha A, Bashtawi M, El-Shanti H. Risk factors for childhood epilepsy: a case-control study from Irbid. Jordan Seizure. 2003;12:171–4. 12651084 10.1016/s1059-1311(02)00194-2

[6] Hauser WA, Annegers JF, Kurland LT. Incidence of epilepsy and unprovoked seizures in Rochester, Minnesota: 1935–1984. Epilepsia. 1993;34:453–68. 8504780 10.1111/j.1528-1157.1993.tb02586.x

[7] Page MJ, McKenzie JE, Bossuyt PM, Boutron I, Hoffmann TC, Mulrow CD, et al. The PRISMA 2020 statement: an updated guideline for reporting systematic reviews. BMJ. 2021;372: n71. 33782057 10.1136/bmj.n71 PMC8005924

[8] Sidik K, Jonkman JN. Robust variance estimation for random effects meta-analysis. Comput Stat Data Anal. 2006;50(12):3681–701.

[9] van Aert RCM, Wicherts JM, van Assen MALM. Publication bias examined in meta-analyses from psychology and medicine: a meta-meta-analysis. PLoS ONE. 2019;14(4):e0215052. 30978228 10.1371/journal.pone.0215052 PMC6461282

[10] Sterne JAC, Harbord RM, Sutton AJ, Jones DR, Ioannidis JP, Terrin N, et al. Recommendations for examining and interpreting funnel plot asymmetry in meta-analyses of randomised controlled trials. BMJ. 2011;343(7818):1–8. 10.1136/bmj.d4002 21784880

[11] Ae-Ngibise KA, Akpalu B, Ngugi A, Akpalu A, Agbokey F, Adjei P, et al. Prevalence and risk factors for active convulsive epilepsy in kintampo. Ghana Pan Afr Med J. 2015;21:29. 26401223 10.11604/pamj.2015.21.29.6084 PMC4561141

[12] Ngugi AK, Bottomley C, Kleinschmidt I, Wagner RG, Kakooza-Mwesige A, Ae-Ngibise K, et al. Prevalence of active convulsive epilepsy in sub-Saharan Africa and associated risk factors: cross-sectional and case-control studies. Lancet Neurol. 2013;12:253–63. 23375964 10.1016/S1474-4422(13)70003-6 PMC3581814

[13] Sun Y, Vestergaard M, Christensen J, Nahmias AJ, Olsen J. Prenatal exposure to maternal infections and epilepsy in childhood: a population-based cohort study. Pediatrics. 2008;121:e1100–7. 18450853 10.1542/peds.2007-2316

[14] Asadi-Pooya AA, Hojabri K. Risk factors for childhood epilepsy: a case-control study. Epilepsy Behav. 2005;6:203–6. 15710305 10.1016/j.yebeh.2004.11.018

[15] Malmqvist O, Ohlin A, Ågren J, Jonsson M. Seizures in newborn infants without hypoxic ischemic encephalopathy - antenatal and labor-related risk factors: a case-control study. J Matern Fetal Neonatal Med. 2020;33:799–805. 30373414 10.1080/14767058.2018.1505853

[16] McDermott S, Mann JR, Wu J. Maternal genitourinary infection appears to synergistically increase the risk of epilepsy in children of women with epilepsy. Neuroepidemiology. 2010;34:117–22. 20051695 10.1159/000268824

[17] Krebs L, Langhoff-Roos J. The relation of breech presentation at term to epilepsy in childhood. Eur J Obstet Gynecol Reprod Biol. 2006;127:26–8. 15950370 10.1016/j.ejogrb.2004.05.018

[18] Hirvonen M, Ojala R, Korhonen P, Haataja P, Eriksson K, Gissler M, et al. The incidence and risk factors of epilepsy in children born preterm: a nationwide register study. Epilepsy Res. 2017;138:32–8. 29054051 10.1016/j.eplepsyres.2017.10.005

[19] Odd D, Glover Williams A, Winter C, Draycott T. Associations between early term and late/post term infants and development of epilepsy: a cohort study. PLoS ONE. 2018;13:e0210181. 30596766 10.1371/journal.pone.0210181 PMC6312375

[20] Whitehead E, Dodds L, Joseph KS, Gordon KE, Wood E, Allen AC, et al. Relation of pregnancy and neonatal factors to subsequent development of childhood epilepsy: a population-based cohort study. Pediatrics. 2006;117:1298–306. 16585327 10.1542/peds.2005-1660

[21] Chou IC, Sung FC, Hong SY. Incidence of epilepsy in children born prematurely and small for gestational age at term gestation: A population-based cohort study. J Paediatr Child Health. 2020;56:324–9. 31464013 10.1111/jpc.14611

[22] Burton KJ, Rogathe J, Whittaker R, Mankad K, Hunter E, Burton MJ, et al. Epilepsy in Tanzanian children: association with perinatal events and other risk factors. Epilepsia. 2012;53(4):752–60. 22308971 10.1111/j.1528-1167.2011.03395.x PMC3467761

[23] Cansu A, Serdaroğlu A, Yüksel D, Doğan V, Ozkan S, Hirfanoğlu T, et al. Prevalence of some risk factors in children with epilepsy compared to their controls. Seizure. 2007;16:338–44. 17391991 10.1016/j.seizure.2007.02.003

[24] Cruz-Cruz MDR, Gallardo-Elías J, Paredes-Solís S, Legorreta-Soberanis J, Flores-Moreno M, Andersson N. Factores asociados a epilepsia en niños en México: un estudio caso-control [Factors associated with epilepsy in children in Mexico: a case-control study]. Bol Med Hosp Infant Mex. 2017;74(5):334–40. 29382476 10.1016/j.bmhimx.2017.05.006

[25] Attumalil TV, Sundaram A, Varghese VO, Vijayakumar K, Kunju PA. Risk factors of childhood epilepsy in Kerala. Ann Indian Acad Neurol. 2011;14:283–6. 22346018 10.4103/0972-2327.91950 PMC3271468

[26] Chowdhury SH, Tabassum R, Tarannum R, Kabir S. Pregnancy related factors associated with epileptic & NonEpileptic children. IOSR J Dent Med Sci. 2020;19:19–25.

[27] Kakooza-Mwesige A, Ndyomugyenyi D, Pariyo G, Peterson SS, Waiswa PM, Galiwango E, et al. Adverse perinatal events, treatment gap, and positive family history linked to the high burden of active convulsive epilepsy in Uganda: a population-based study. Epilepsia Open. 2017;2:188–98. 29588948 10.1002/epi4.12048 PMC5719853

[28] Masri A, Badran E, Hamamy H, Assaf A, Al-Qudah AA. Etiologies, outcomes, and risk factors for epilepsy in infants: a case-control study. Clin Neurol Neurosurg. 2008;110(4):352–6. 18249488 10.1016/j.clineuro.2007.12.013

[29] Mung’ala-Odera V, White S, Meehan R, Otieno GO, Njuguna P, Mturi N, et al. Prevalence, incidence and risk factors of epilepsy in older children in rural Kenya. Seizure. 2008;17(5):396–404. 18249012 10.1016/j.seizure.2007.11.028 PMC3428880

[30] Kuenneth CA, Boyle C, Murphy CC, Yeargin-Allsopp M. Reproductive risk factors for epilepsy among ten-year-old children in metropolitan Atlanta. Paediatr Perinat Epidemiol. 1996;10:186–96. 8778691 10.1111/j.1365-3016.1996.tb00042.x

[31] Maheshwari MC. Forceps delivery as a risk factor in epilepsy: a comparative prospective cohort survey. Acta Neurol Scand. 1990;81:522–3. 2220310 10.1111/j.1600-0404.1990.tb01012.x

[32] Henderson M, Goldstein H, Rogot E, Goldberg ID, Entwisle G. Perinatal factors associated with epilepsy in Negro children. Public Health Rep. 1964;79:501–9. PMC1915453 14155848

[33] Gumisiriza N, Kugler M, Brusselaers N, Mubiru F, Anguzu R, Ningwa A, et al. Risk factors for nodding syndrome and other forms of epilepsy in Northern Uganda: a case-control study. Pathogens. 2021;10(11):1451. 34832607 10.3390/pathogens10111451 PMC8621683

[34] Thygesen SK, Olsen M, Pedersen L, Henderson VW, Østergaard JR, Sørensen HT. Respiratory distress syndrome in preterm infants and risk of epilepsy in a Danish cohort. Eur J Epidemiol. 2018;33:313–21. 28887607 10.1007/s10654-017-0308-1

[35] Li W, Peng A, Deng S, Lai W, Qiu X, Zhang L, et al. Do premature and postterm birth increase the risk of epilepsy? An updated meta-analysis. Epilepsy Behav. 2019;97:83–91. 31202097 10.1016/j.yebeh.2019.05.016

[36] Berger R, Garnier Y, Jensen A. Perinatal brain damage: underlying mechanisms and neuroprotective strategies. J Soc Gynecol Investig. 2002;9:319–28. 12445595

[37] Berg A, Nickels K, Wirrell E, Geerts A, Callenbach P, Arts F, et al. Mortality risks in new-onset childhood epilepsy. Pediatrics. 2013;132:124–31. 23753097 10.1542/peds.2012-3998 PMC3691537

[38] Guan H, Zhou P, Qi Y, Huang H, Wang J, Liu X. Cigarette smoke-induced trophoblast cell ferroptosis in rat placenta and the effects of L-arginine intervention. Ecotoxicol Environ Saf. 2022;243:114015. 36030684 10.1016/j.ecoenv.2022.114015

[39] Zdravkovic T, Genbacev O, McMaster MT, Fisher SJ. The adverse effects of maternal smoking on the human placenta: a review. Placenta. 2005;26:S81–6. 15837073 10.1016/j.placenta.2005.02.003

[40] Rong L, Frontera AT Jr, Benbadis SR. Tobacco smoking, epilepsy, and seizures. Epilepsy Behav. 2014;31:210–8. 24441294 10.1016/j.yebeh.2013.11.022

[41] Ekblad M, Korkeila J, Parkkola R, Lapinleimu H, Haataja L, Lehtonen L, et al. Maternal smoking during pregnancy and regional brain volumes in preterm infants. J Pediatr. 2010;156(2):185–90. 19818449 10.1016/j.jpeds.2009.07.061

[42] Falk L, Nordberg A, Seiger A, Kjaeldgaard A, Hellstrom-Lindahl E. Smoking during early pregnancy affects the expression pattern of both nicotinic and muscarinic acetylcholine receptors in human first trimester brainstem and cerebellum. Neuroscience. 2005;132:389–97. 15802191 10.1016/j.neuroscience.2004.12.049

[43] Dreier JW, Ellis CA, Berkovic SF, Cotsapas C, Ottman R, Christensen J. Epilepsy risk in offspring of affected parents; a cohort study of the “maternal effect” in epilepsy. Ann Clin Transl Neurol. 2021;8(1):153–62. 33249752 10.1002/acn3.51258 PMC7818075

[44] Berkovic SF, Scheffer IE. Genetics of the epilepsies. Epilepsia. 2001;42:16–23. 11887962 10.1046/j.1528-1157.2001.0420s5016.x

[45] Rocca WA, Sharbrough FW, Hauser WA, Annegers JF, Schoenberg BS. Risk factors for absence seizures: a population-based case-control study in Rochester. Minnesota Neurology. 1987;37(8):1309–14. 3112608 10.1212/wnl.37.8.1309

[46] Casetta I, Monetti VC, Malagù S, Paolino E, Govoni V, Fainardi E, et al. Risk factors for cryptogenic and idiopathic partial epilepsy: a community-based case-control study in Copparo. Italy Neuroepidemiology. 2002;21(5):251–4. 12207154 10.1159/000065644

[47] Glass HC, Pham TN, Danielsen B, Towner D, Glidden D, Wu YW. Antenatal and intrapartum risk factors for seizures in term newborns: a population-based study, California 1998–2002. J Pediatr. 2009;154(1):24–8. 18760807 10.1016/j.jpeds.2008.07.008 PMC2635430

[48] Kieslich M, Jacobi G. Incidence and risk factors of posttraumatic epilepsy in childhood. Lancet. 1995;345(21):187. 7823678 10.1016/s0140-6736(95)90188-4

[49] Serdaroğlu A, Ozkan S, Aydin K, Gücüyener K, Tezcan S, Aycan S. Prevalence of epilepsy in Turkish children between the ages of 0 and 16 years. J Child Neurol. 2004;19(4):271–4. 15163093 10.1177/088307380401900406

[50] Reddy DS, Thompson W, Calderara G. Molecular mechanisms of sex differences in epilepsy and seizure susceptibility in chemical, genetic and acquired epileptogenesis. Neurosci Lett. 2021;750:135753. 33610673 10.1016/j.neulet.2021.135753 PMC7994197

[51] Savic I, Engel J Jr. Structural and functional correlates of epileptogenesis - does gender matter? Neurobiol Dis. 2014;70:69–73. 24943053 10.1016/j.nbd.2014.05.028 PMC5003532

[52] Lenz KM, McCarthy MM. A starring role for microglia in brain sex differences. Neuroscientist. 2015;21(3):306–21. 24871624 10.1177/1073858414536468 PMC5742269

[53] Morizawa Y, Sato K, Takaki J, Kawasaki A, Shibata K, Suzuki T, et al. Cell-autonomous enhancement of glutamate-uptake by female astrocytes. Cell Mol Neurobiol. 2012;32(6):953–6. 22450870 10.1007/s10571-012-9829-z PMC11498379

[54] Luders E, Gaser C, Narr KL, Toga AW. Why sex matters: brain size independent differences in gray matter distributions between men and women. J Neurosci. 2009;29(45):14265–70. 19906974 10.1523/JNEUROSCI.2261-09.2009 PMC3110817

[55] McGrath MM, Sullivan MC, Lester BM, Oh W. Longitudinal neurologic follow-up in neonatal intensive care unit survivors with various neonatal morbidities. Pediatrics. 2000;106(6):1397–405. 11099595 10.1542/peds.106.6.1397

[56] Graham HK, Rosenbaum P, Paneth N, Dan B, Lin JP, Damiano DL, et al. Cerebral palsy. Nat Rev Dis Primers. 2016;2:15082. 27188686 10.1038/nrdp.2015.82 PMC9619297

[57] Ahlers FS, Benros ME, Dreier JW, Christensen J. Infections and risk of epilepsy in children and young adults: a nationwide study. Epilepsia. 2019;60(2):275–83. 30577081 10.1111/epi.14626

[58] Vezzani A, Fujinami RS, White HS, Preux PM, Blümcke I, Sander JW, et al. Infections, inflammation and epilepsy. Acta Neuropathol. 2016;131(2):211–34. 26423537 10.1007/s00401-015-1481-5 PMC4867498

[59] Yang G, Zou LP, Wang J, Shi X, Tian S, Yang X, et al. Neonatal hypoglycemic brain injury is a cause of infantile spasms. Exp Ther Med. 2016;11(5):2066–70. 27168852 10.3892/etm.2016.3107 PMC4840632

[60] Scher MS. Prenatal contributions to epilepsy: lessons from the bedside. Epileptic Disord. 2003;5(2):77–91. 12875951

[61] Watila MM, Balarabe SA, Komolafe MA, Igwe SC, Fawale MB, Otte WM, et al. Epidemiology of epilepsy in nigeria: a community-based study from 3 sites. Neurology. 2021;97(7):e728–38. 34253632 10.1212/WNL.0000000000012416

